# Supplementary material for: Resolving the full spectrum of human genome variation using Linked-Reads
Source: Genome Res. 2019 Apr;29(4):635–45. doi: 10.1101/gr.234443.118 (PMC6442396; doi:10.1101/gr.234443.118)
Supplement: Supplemental Material [file supp_gr.234443.118_Supplemental_longranger-2.2.zip › longranger-2.2.tar.bz2!/lr2.2-public/lib/stan/cmdstan/stan/doc/api/html/dir_34177b290fffdbdb584997ce852f13d5.html]

Stan: src/stan/common Directory Reference


|  |  |
| --- | --- |
|  | Stan  2.5.0  probability, sampling & optimization |


- Main Page
- Related Pages
- Namespaces
- Classes
- Files

All Classes Namespaces Files Functions Variables Typedefs Enumerations Enumerator Friends Macros Pages

- src
- stan
- common

common Directory Reference

|  |  |
| --- | --- |
| Directories | |
| directory | recorder |
|  | |

|  |  |
| --- | --- |
| Files | |
| file | command.hpp [code] |
|  | |
| file | context\_factory.hpp [code] |
|  | |
| file | do\_bfgs\_optimize.hpp [code] |
|  | |
| file | do\_print.hpp [code] |
|  | |
| file | init\_adapt.hpp [code] |
|  | |
| file | init\_nuts.hpp [code] |
|  | |
| file | init\_static\_hmc.hpp [code] |
|  | |
| file | init\_windowed\_adapt.hpp [code] |
|  | |
| file | initialize\_state.hpp [code] |
|  | |
| file | print\_progress.hpp [code] |
|  | |
| file | recorder.hpp [code] |
|  | |
| file | run\_markov\_chain.hpp [code] |
|  | |
| file | sample.hpp [code] |
|  | |
| file | warmup.hpp [code] |
|  | |
| file | write\_error\_msg.hpp [code] |
|  | |
| file | write\_iteration.hpp [code] |
|  | |
| file | write\_iteration\_csv.hpp [code] |
|  | |
| file | write\_model.hpp [code] |
|  | |
| file | write\_stan.hpp [code] |
|  | |


---

[ Stan Home Page ]

*© 2011–2014,
Stan Development Team.*
